# The prospects of selection for social genetic effects to improve welfare and productivity in livestock

**DOI:** 10.3389/fgene.2014.00377

**Published:** 2014-11-11

**Authors:** Esther D. Ellen, T. Bas Rodenburg, Gerard A. A. Albers, J. Elizabeth Bolhuis, Irene Camerlink, Naomi Duijvesteijn, Egbert F. Knol, William M. Muir, Katrijn Peeters, Inonge Reimert, Ewa Sell-Kubiak, Johan A. M. van Arendonk, Jeroen Visscher, Piter Bijma

**Affiliations:** ^1^Animal Breeding and Genomics Centre, Wageningen UniversityWageningen, Netherlands; ^2^Behavioural Ecology Group, Wageningen UniversityWageningen, Netherlands; ^3^Hendrix Genetics, Research and Technology CentreBoxmeer, Netherlands; ^4^Adaptation Physiology Group, Wageningen UniversityWageningen, Netherlands; ^5^TOPIGS Research Centre IPGBeuningen, Netherlands; ^6^Department of Animal Science, Purdue UniversityWest Lafayette, IN, USA; ^7^Institut de Sélection AnimaleBoxmeer, Netherlands

**Keywords:** genetic selection, social genetic effects, welfare, laying hens, pigs

## Abstract

Social interactions between individuals living in a group can have both positive and negative effects on welfare, productivity, and health of these individuals. Negative effects of social interactions in livestock are easier to observe than positive effects. For example, laying hens may develop feather pecking, which can cause mortality due to cannibalism, and pigs may develop tail biting or excessive aggression. Several studies have shown that social interactions affect the genetic variation in a trait. Genetic improvement of socially-affected traits, however, has proven to be difficult until relatively recently. The use of classical selection methods, like individual selection, may result in selection responses opposite to expected, because these methods neglect the effect of an individual on its group mates (social genetic effects). It has become clear that improvement of socially-affected traits requires selection methods that take into account not only the direct effect of an individual on its own phenotype but also the social genetic effects, also known as indirect genetic effects, of an individual on the phenotypes of its group mates. Here, we review the theoretical and empirical work on social genetic effects, with a focus on livestock. First, we present the theory of social genetic effects. Subsequently, we evaluate the evidence for social genetic effects in livestock and other species, by reviewing estimates of genetic parameters for direct and social genetic effects. Then we describe the results of different selection experiments. Finally, we discuss issues concerning the implementation of social genetic effects in livestock breeding programs. This review demonstrates that selection for socially-affected traits, using methods that target both the direct and social genetic effects, is a promising, but sometimes difficult to use in practice, tool to simultaneously improve production and welfare in livestock.

## Introduction

Social interactions among individuals can have large effects on their phenotypes, both in domestic and natural populations. Such interactions affect the outcome of evolutionary processes and of domestic breeding programs (e.g., Hamilton, 1964; Griffing, 1967, 1977; Frank, 1998; Denison et al., 2003; Muir, 2005; Rodenburg et al., 2010; Bijma, 2011a). Social interactions can have both positive and negative effects on welfare, productivity, and health of livestock. Cooperation and mothering behavior are examples of positive social interactions, whereas competition and aggression are examples of negative social interactions.

There are more examples of negative than of positive social interactions in livestock. Domestic laying hens, for example, can develop feather pecking and cannibalism, which may result in mortality (Allen and Perry, 1975; Blokhuis and Arkes, 1984; Savory, 1995; Craig and Muir, 1996; Kjaer and Sørensen, 1997; Rodenburg et al., 2013). Domestic pigs may show injurious behaviors such as tail biting (Schrøder-Petersen and Simonsen, 2001; Zupan et al., 2012). Using classical selection methods, such as mass selection or selection on estimated breeding values, animal breeders have successfully improved many traits of agricultural importance. Typical examples are growth rate in broilers and pigs and egg number in laying hens (Hill, 2008). Genetic selection can also be used to improve traits affected by social interactions (in this review we will refer to these traits as socially-affected traits) and, thereby, reduce the negative effects of social interactions in livestock. With classical selection methods, however, improvement of socially-affected traits has been proven to be difficult (Wade, 1976, 1977; Goodnight, 1985; Craig and Muir, 1996; Muir and Cheng, 2004). Those selection methods target only the direct effect of an individual's genotype on its own phenotype and neglect the social effect of an individual on the phenotype of other individuals (Griffing, 1967). The use of classical selection methods for socially-affected traits has sometimes resulted in responses in the opposite direction (Wade, 1976, 1977; Craig and Muir, 1996; Muir, 2005). This can occur because the best individuals in the classical setting may have negative genetic effects on other individuals. For example, laying hens that have good genes for survival could also be more likely to show high levels of aggressive and competitive behavior. The use of such hens as parents for the next generation reduces survival of their group mates and potentially of the entire population (Muir and Cheng, 2004).

At first glance, selection for improved social behaviors should ideally be based on behavioral observations. In laying hens, for example, number of bouts of feather pecking was used to select against feather pecking behavior. After three generations, feather pecking was significantly decreased (Kjaer et al., 2001). Unfortunately, collecting behavioral observations is very time consuming, making breeding based on behavioral observations not feasible in practice. Moreover, individual behavior may depend not only on the genotype of the individual expressing the behavior, but also on the genotype of its social partners. Cannibalism in laying hens, for example, depends both on a genetic effect due to the actor (the pecker), and a genetic effect originating from the victim (Ellen et al., 2008). Simply selecting against pecking behavior using behavioral observations will disregard the genetic variation originating from the victim, and therefore yield a suboptimal response. Thus, breeding based on behavioral observations both requires an unrealistic effort with respect to data collection, and disregards part of the genetic variation. Breeders, therefore, need better solutions.

A solution feasible in practice may come from statistical methods that take into account both the direct genetic effect of an individual on its own phenotype and the social genetic effect of an individual on the phenotype of its group mates [also known as associative effect or Indirect Genetic Effect (IGE)]. Such methods allow us to estimate both the breeding value for the direct effect and the breeding value for the social effect, without the need for behavioral observations. For mortality due to cannibalism in laying hens, for example, the direct effect corresponds to the victim effect, whereas the social effect corresponds to the pecker effect (Ellen et al., 2008; Peeters et al., 2012). The advantage of such methods is that they capture the total genetic variation underlying the trait (Bijma, 2011b; see below).

Here, we review the theoretical and empirical work on social genetic effects, with a focus on livestock. First, we present the theory of social genetic effects. Subsequently, we evaluate the evidence for social genetic effects in livestock and other species, by reviewing estimates of genetic parameters for direct and social genetic effects. Then we describe the results of different selection experiments. Finally, we discuss issues concerning the implementation of social genetic effects in livestock breeding programs.

## Theoretical background

Models of socially-affected traits have been developed within two frameworks (McGlothlin and Brodie, 2009; Bijma, 2014). In the so-called trait-based framework (Moore et al., 1997; Wolf et al., 1998), the social effect of a focal individual on trait values of other individuals is modeled as a function of specific traits of the focal individual. Hence, trait-based models describe the social effect as a function of observable traits and explicitly model the mechanism underlying the social effect. Trait-based models of social effects are an extension of maternal-effect models of Falconer (1965) and Kirkpatrick and Lande (1989). In the variance-component framework, in contrast, the traits causing the social effects are not specified. Instead, the social effect is added to the model as an additional random genetic effect (Griffing, 1967, 1977, 1981), similar to the maternal-genetic effects models of Willham (1963), and its variance is estimated based on family relationships in the data. Trait-based models may be of greater biological interest as they provide insight in the traits underlying the social effects, whereas variance-component models are empirically more powerful because they can be applied without knowledge of those traits. For livestock genetic improvement, the variance-component models are more relevant, because the traits underlying the social effects are usually unknown and recording of a wide range of traits on individuals is difficult. The following, therefore, considers only variance-component models.

### Model

In the classical quantitative genetic model (Fisher, 1918), the phenotype of an individual is the sum of its breeding value and a residual non-heritable effect (*P* = *A* + *E*). With social interactions, the model needs to be extended to incorporate social effects. When social interactions occur within a group consisting of *n* individuals, the phenotype of individual *i* may be modeled as the sum of its own direct effect, and the sum of the social effects of each of its *n* − 1 group mates. Both the direct and social effect can be partitioned into an additive genetic and a non-heritable (residual) component (Griffing, 1967),

(1) $$P_{i}=\;A_{D,i}+E_{D,i}+\sum_{j\;\neq \;i}^{n\;-\;1}\left(A_{S,j}+E_{S,j}\right)$$

where *A*_*D*,*i*_ is the direct breeding value (DBV; see Table 1 for notation) of individual *i*, *E*_*D*,*i*_ is the corresponding non-heritable direct effect, *A*_*S*,*j*_ the social breeding value (SBV) of group member *j*, and *E*_*S*,*j*_ the corresponding non-heritable social effect. This model applies to each of the *n* group members. Note that DBV and SBV are distinct breeding values. For example, when the trait of interest is survival, the DBV refers to the heritable effect of an individual on its own survival, whereas the SBV refers to the heritable effect of an individual on survival of its group mates, which may, for example, relate to aggression. So the DBV is comparable to the “classical” breeding value (Lynch and Walsh, 1998), whereas the SBV is a generalization of a breeding value for a maternal effect (Willham, 1963).

**Table 1:** **Notation key**.

**Symbol**	**Meaning**
*P*_*i*_	Observed trait value for individual *i*
j, n	Group mate of individual *i*, Group size
*A*_*D*,*i*_, *A*_*S*,*i*_	Direct genetic effect of *i*, social genetic effect of *i*
*E*_*D*,*i*_, *E*_*S*,*i*_	Direct non-genetic effect of *i*, social non-genetic effect of *i*
DBV, SBV	Direct breeding value, social breeding value
TBV*_i_*	Total breeding value of *i*: *TBV_i_* = *A_D,i_* + (*n* − 1) *A_S,i_*
σ^2^_*A*_*D*__, σ^2^_*A*_*S*__	Direct genetic variance, social genetic variance
σ_*A*_*DS*__, *r*_*A*_	Covariance and correlation between direct and social genetic effects
σ^2^_*TBV*_, *T*^2^	Total heritable variance, relative heritable variance
σ^2^_*P*_	Phenotypic variance
σ_*P*_, σ_*P*_*grp*__ σ_*P*_*rel*__	Standard deviation among phenotypic values of individuals, among average phenotypic values of groups, and among average phenotypic values of relatives in family groups
σ^2^_*SC*_	Variance of the selection criterion
Δ*G*	Selection response in observed trait value per generation
SC*_i_*	Selection criterion
ι, ρ	Selection intensity, accuracy of selection
*r*	Relatedness between selection candidates and its relatives
*r*_*rel*_	Relatedness between group members
g	Degree of between-group selection
τ	Intraclass correlation among relatives adjusted for interactions
η^2^	Analogy of heritability: σ^2^_*TBV*_/σ^2^_*TPV*_

In populations consisting of groups of *n* members, each individual expresses its DBV once in its own phenotype and its SBV *n* − 1 times, once in the phenotypes of each of its *n*−1 group mates. The total heritable impact of a single individual's genes on the mean trait value of the population is, therefore, given by the individual's total breeding value (TBV; Moore et al., 1997; Muir, 2005; Bijma et al., 2007b),

(2) $$TBV_{i}=A_{D,i}+\left(n-1\right)A_{S,i}$$

Note that, in contrast to the phenotype (Equation 1), the TBV in Equation 2 is entirely a heritable property of individual *i* itself. It is a generalization of the classical breeding value, and is the heritable component relevant for response to selection in socially-affected traits (Bijma, 2011b). The total heritable variance available for response to selection equals the variance in TBVs among individuals (Griffing, 1977; Bijma et al., 2007a),

(3) $$\sigma_{TBV}^{2}=\sigma_{A_{D}}^{2}+2\left(n-1\right)\sigma_{A_{DS}}+{\left(n-1\right)}^{2}\sigma_{A_{S}}^{2}$$

where σ^2^_*A*_*D*__ is the direct genetic variance, σ^2^_*A*_*S*__ is the social genetic variance, and σ_*A*_*DS*__ is the covariance between DBVs and SBVs of individuals. The direct-social genetic covariance indicates the relationship between the direct and social effects expressed by an individual. For example, if individuals that show cannibalistic behavior have on average better survival themselves, then the direct-social genetic covariance is negative. The magnitude of social effects may depend on group size, and for most traits it is probably smaller in larger groups. This is relevant for the estimation of social effects from data with varying group size, and also for the relationship of total heritable variance and response to selection with group size. The dependency of social effects on group size can be modeled as a dilution effect (Arango et al., 2005; Bijma, 2010b). For details see Bijma (2010b).

Analogous to ordinary heritability, the total heritable variance can be expressed relative to the phenotypic variance (Bergsma et al., 2008),

(4) $$T^{2}=\frac{\sigma_{TBV}^{2}}{\sigma_{P}^{2}}$$

A comparison between *T*^2^ and classical heritability reveals the impact of social interactions on the heritable variation that determines the potential of the population to respond to selection.

### Selection response

The classical expression for response to selection is the product of the intensity of selection, ι, the accuracy of selection, ρ, and the additive genetic standard deviation, σ_*A*_; Δ*G* = ιρσ_*A*_. This expression can be generalized to encompass socially-affected traits (Griffing, 1977; Ellen et al., 2007; Wade et al., 2010),

(5) $$\Delta G=\iota \rho_{TBV}\sigma_{TBV}.$$

The σ_*TBV*_ is the square root of total heritable variance (Equation 3) and ρ_*TBV*_ is the accuracy which is the correlation between the selection criterion and the total breeding value in the selection candidates (Bijma, 2011a). The accuracy is the key parameter measuring the quality of a selection criterion. The following shows that relatedness between interacting individuals is the most important factor determining the accuracy for socially-affected traits.

### Accuracy of selection

Below we describe five different selection methods that can be applied to improve socially-affected traits; individual selection, group selection, multilevel selection, selection based on relatives, and selection on estimated breeding values. With the first three methods, selection candidates need to be kept in groups, whereas with the last two methods selection candidates can be kept individually and can be selected based on information from group-housed relatives. For each of the five selection methods, we present expressions for accuracy of selection. Derivations are given in Griffing (1977), Ellen et al. (2007), Bijma and Wade (2008), Wade et al. (2010), and Bijma (2011a). Table 2 summarizes the selection methods, the selection criteria, and the accuracies.

**Table 2:** **Selection criterion and accuracies of the different selection methods**.

**Selection method^a^**	**Selection criterion**	**Accuracy^b^**
IS	*P*_*i*_	$\left\{r\sigma_{TBV}^{2}+\left(1-r\right)\left[\sigma_{A_{D}}^{2}+\left(n-1\right)\sigma_{A_{DS}}\right]\right\}/\sigma_{P}\sigma_{TBV}$
GS	*P*_*grp*_	[(*n* − 1)*r* + 1]σ_*TBV*_/*n*σ_*P*_*grp*__
MS	*P_i_* + *g* · ∑_*n* − 1_ *P_j_*	$\frac{\left[g+r+\left(n-2\right)gr\right]\sigma_{TBV}^{2}+\left(1-g\right)\left(1-r\right)\left[\sigma_{A_{D}}^{2}+\left(n-1\right)\sigma_{A_{DS}}\right]}{\sigma_{TBV}\sigma_{SC}}$
SR	*P*_*rel*_*i*__ = 1/*mn*∑^*m*^_*l* = 1_ ∑*^n^*_*j* = 1_*P*_*j*,*l*_	$r_{rel}\eta /\sqrt{\tau +\left(1-\tau \right)/mn}where\;\eta =\sigma_{TBV}/\sigma_{TPV},\;\tau =r\eta^{2}$
EBV	$\hat{a}$*_i_*	$\approx \rho_{MME}\left[\frac{\sigma_{A_{D}}^{2}+\left(n-1\right)\sigma_{A_{DS}}}{\sigma_{A_{D}}\sigma_{TBV}}\right]$

a IS is individual selection; GS is group selection; MS is multilevel selection; SR is selection based on relatives; EBV is selection on estimated breeding values ignoring social genetic effects;

b *r denotes relatedness between group members; n = number of group members; mn = number of relatives in m groups; r_rel_ = relatedness between the candidate and its relatives; σ^2^_TBV_ = σ^2^_A_D__ + 2(n − 1)σ_A_DS__ + (n − 1)^2^σ^2^_A_S__; σ^2^_P_ = σ^2^_A_D__ + σ^2^_E_D__ + (n − 1)(σ^2^_A_S__ + σ^2^_E_S__) + r[2(n − 1)σ_A_DS__ + (n − 1)(n − 2)σ^2^_A_S__]; σ^2^_P_grp__ = {σ^2^_P_ + 2(n − 1) Cov(P_i_, P_j_) + (n − 1)[σ^2^_P_ + (n − 2) Cov(P_i_, P_j_)]}/n^2^ (Ellen et al., 2007); σ^2^_SC_ = σ^2^_P_ + 2gCov (P,P_grp_) + g^2^σ^2^_P_grp__ (Bijma and Wade, 2008; Wade et al., 2010); σ^2^_TPV_ = σ^2^_P_D__ + 2(n − 1)σ_P_DS__ + (n − 1)^2^σ^2^_P_S__*.

#### Individual selection (IS)

With individual or mass selection, group-housed selection candidates with the best phenotypes are selected as parents of the next generation. Thus, the selection criterion is the individual trait value, *SC_i_* = *P_i_* (Wade et al., 2010). Accuracy of individual selection equals (Wade et al., 2010)

(6) $$\rho_{TBV,IS}=\frac{r\sigma_{TBV}^{2}+\left(1-r\right)\left[\sigma_{A_{D}}^{2}+\left(n-1\right)\sigma_{A_{DS}}\right]}{\sigma_{TBV}\sigma_{P}}$$

In the numerator of this expression, the first term is always positive, whereas the second term can take negative values when the direct-social genetic covariance is sufficiently negative. When group members are unrelated (*r* = 0), accuracy depends only on the second term in the numerator, and can thus be negative when direct and social genetic effects are negatively correlated (Griffing, 1967, 1977). This theoretical prediction agrees with empirical observations (Wade, 1976; Craig, 1982; Goodnight, 1985; Agrawal et al., 2001; Muir, 2005; Muir et al., 2013). In *Tribolium*, for example, it was found that individual selection for increased population size gave a decrease in population size in the next generation (Wade, 1976). Muir (2005), Muir et al. (2013) showed in quail selected for 6-week body weight in groups of 16, that individual selection in unrelated groups resulted in a slight decline. With unrelated group members, therefore, individual or mass selection is inadequate to improve socially-affected traits. With fully related group members (*r* = 1, i.e., clones), accuracy is always positive so that response is in the same direction as selection. However, usually a limited relatedness suffices to guarantee positive accuracy (Wade et al., 2010).

#### Group selection (GS)

With group selection, groups with the highest average phenotypic value are selected to become parents of the next generation (Muir, 1996). Thus, the selection criterion is the group average, *SC_i_* = *P_grp_*. Accuracy of group selection equals (Ellen et al., 2007)

(7) $$\rho_{TBV,GS}=\frac{\left[\left(n-1\right)r+1\right]\sigma_{TBV}}{n\sigma_{{\bar{P}}_{grp}}}$$

where σ*_P__grp_* denotes the standard deviation in the average phenotype of group members. In equation 7, both the numerator and denominator are positive, which results in a positive accuracy and a positive response to selection. Thus, group selection prevents negative response to selection. Group selection is, however, only efficient when group members are sufficiently related (Bijma, 2011a). As shown by Muir (1996), group selection can result in rapid short-term responses. However, when groups are composed of relatives, selection between groups will result in between-family selection, which increases rates of inbreeding (Muir et al., 2013). Hence, this selection method should be combined with selection algorithms that restrict the rate of inbreeding, such as optimal contribution selection (Meuwissen, 1997).

#### Multilevel selection (MS)

With multilevel selection, selection is based on a linear combination of the phenotypes of the individual and the phenotype of its group mates, *SC_i_* = *P_i_* + *g* · ∑_*n* − 1_ *P_j_*, where *g* is the degree of group selection (*g* = 0 corresponds to individual selection, whereas *g* = 1 corresponds to group selection) (Griffing, 1977; Bijma et al., 2007b; Muir et al., 2013). The accuracy of multilevel selection equals (Wade et al., 2010)

(8) $$\rho_{TBV,MS}=\frac{\begin{array}{c} \{\left[g+r+\left(n-2\right)gr\right]\sigma_{TBV}^{2}+\\ \left(1-g\right)\left(1-r\right)\left[\sigma_{A_{D}}^{2}+\left(n-1\right)\sigma_{A_{DS}}\right]\} \end{array}}{\sigma_{TBV}\sigma_{SC}}$$

where σ^2^_*SC*_ is the variance of the selection criterion. Equation 8 shows that both multilevel selection (*g* > 0) and relatedness between group mates (*r* > 0) create a positive accuracy, so that response to selection is positive. Without multilevel selection (*g* = 0), Equation 8 reduces to Equation 6.

#### Selection based on relatives (SR)

The above three selection methods have considered selection candidates kept in groups. Keeping selection candidates in groups, however, may be undesirable because it may interfere with collection of individual trait values, such as egg number in laying hens. To improve socially-affected traits when selection candidates are kept individually, information of relatives kept in family groups can be used (Ellen et al., 2007). In such schemes, individually housed selection candidates are selected based on the performance of sib or offspring groups, *SC_i_* = *P_rel,i_* (Ellen et al., 2007). Keeping relatives in family groups guarantees that both direct and social effects are captured in the selection criterion, even when social effects are ignored in the breeding value estimation (e.g., because genetic parameters are unknown). When relatives are kept in *m* groups of *n* individuals each, the accuracy of selection based on relatives (Ellen et al., 2007) equals

(9) $$\rho_{TBV,SR}=\frac{r_{rel}\eta }{\sqrt{\tau +\left(1-\tau \right)/mn}},$$

in which τ = *r*η^2^, being the intraclass correlation between relatives; η = σ*_TBV_*/σ*_TPV_* is an analogy of the square root of heritability; and *mn* is the number of relatives for each selection candidate (*m* is number of groups with *n* relatives each). Ellen et al. (2007) showed that using full sib groups (either full sibs of the selection candidate, or full-sib offspring of the selection candidate) gave the highest accuracies, and thus the highest expected responses to selection. Particularly when relatives are sibs of the selection candidates, restriction of the rate of inbreeding requires attention.

#### Selection on estimated breeding values (EBV)

For the above selection methods, knowledge of genetic parameters is not needed. When genetic parameters of a trait are known, however, the use of BLUP (Best Linear Unbiased Prediction) to estimate breeding values is to be preferred, because it utilizes information of all relatives and corrects for systematic environmental effects, such as herd-year-season effects (Henderson, 1975). Often genetic parameters for ordinary (direct) breeding values will be known, but parameters for the social effects may not be known. In that case, BLUP may be implemented ignoring social genetic effects. In the following, therefore, we will first consider selection on BLUP-EBV when social effects are ignored, and subsequently consider the case where social effects are included in the model.

***Ignoring social genetic effects (EDBV)***. In this case, breeding values are predicted using the classical mixed animal model

(10) y=Xb+Za+e,

where **y** is the vector of observations, **b** is a vector of fixed effects with incidence matrix **X**, **a** is a vector of breeding values with incidence matrix **Z** linking phenotypes of individuals to their own breeding value, and **e** is a vector of residuals. Subsequently, animals are selected on their estimated breeding value, *SC_i_* = $\hat{a}$_*i*_. When group members are unrelated, the approximate accuracy of the classical BLUP approach equals (Bijma, 2011a)

(11) $$\rho_{TBV,BLUP}\left(r=0\right)\approx {\hat{\rho }}_{MME}\left[\frac{\sigma_{A_{D}}^{2}+\left(n-1\right)\sigma_{A_{DS}}}{\sigma_{A_{D}}\sigma_{TBV}}\right],$$

where $\hat{\rho }$_*MME*_ is the ordinary accuracy calculated from the MME, and the term in square brackets is the correlation between an individual's DBV and its TBV. This second term is required because the model predicts the DBV, whereas accuracy of interest is the correlation between EBV and TBV. Thus, using selection for classical BLUP-EBVs with unrelated group members can result in a negative accuracy [when σ^2^*_A_D__* + (n − 1)σ*_A_DS__* < 0], just as with individual selection (Equation 6). When groups are composed of families, however, the EBV resulting from Equation 10 is an estimate of TBV of the individuals; not of their DBV (Bijma, 2011a; Peeters et al., 2013). Hence, in that case the accuracy will always be positive, and ρ*_TBV,BLUP,fam_* ≈ $\hat{\rho }$_*MME*_. This theoretical expectation was confirmed in a selection experiment with quail, where selection for classical BLUP-EBVs with family groups yielded positive response, whereas selection for classical BLUP-EBVs with random groups yielded negative response (Muir et al., 2013).

***Including social genetic effects***. When genetic parameters are known for both direct and social genetic effects, breeding values can be estimated using a direct-indirect effects model (Muir and Schinckel, 2002; Muir, 2005; Muir et al., 2013),

(12) $$y=Xb+Z_{D}\;a_{D}+\;Z_{S}\;a_{S}+Vg+e$$

where **y** is the vector for observations, **b** is a vector of fixed effects with incidence matrix **X**, **a**_*D*_ is a vector of direct breeding values with incidence matrix **Z**_*D*_ linking phenotypes of individuals to their own direct breeding value, **a**_*S*_ is a vector of social breeding values with incidence matrix **Z**_*S*_ linking phenotypes of individuals to the social breeding values of their group mates, **g** is a vector of non-genetic random group effects with incidence matrix **V** (Bergsma et al., 2008), and **e** is a vector of residuals. The covariance structure of the genetic terms is $var\left[\begin{array}{c} a_{D}\\ a_{S} \end{array}\right]=C⊗A$, where $C=\;\left[\begin{array}{cc} \sigma_{A_{D}}^{2} & \sigma_{A_{DS}}\\ \sigma_{A_{DS}} & \sigma_{A_{S}}^{2} \end{array}\right]$, **A** is a matrix of relatedness coefficients between individuals, and ⊗ denotes the Kronecker product of matrices. This model yields estimates of direct and social breeding values, which can be combined into an estimate of the total breeding value, $\hat{a}$_*TBV,i*_ = $\hat{a}$_*D,i*_ + (*n* − 1)$\hat{a}$_*S,i*_, which is the selection criterion; *SC_i_* = $\hat{a}$_*TBV,i*_.

When genetic parameters are known, breeding values can be estimated from the mixed model in Equation 12 irrespective of relatedness among group members. Muir et al. (2010, 2013), however, showed that relatedness within a group resulted in substantially higher accuracy, and that using related group members contributed more to accuracy than distinguishing between direct and social effects in the mixed model (i.e., the use of Equation 12 rather than 10).

#### Predicted responses

To illustrate the results of the different selection methods, we calculated predicted response to selection for survival time in laying hens showing cannibalism. For this trait, accurate genetic parameters have been published, both for purebred (Ellen et al., 2008) and crossbred populations (Peeters et al., 2012). Estimated genetic parameters are shown in Table S1. Predicted responses were calculated from Equation 5, using a selection intensity of unity (ι = 1). For the accuracy, equations presented in Table 2 were used. For the calculation of accuracy, different group compositions were used. Group members were either unrelated (*r* = 0), half sibs (*r* = 0.25), or full sibs (*r* = 0.5). For selection based on relatives, the relationship between selection candidates and relatives kept in groups was either half sibs (*r_rel_* = 0.25) or full sibs (*r_rel_* = 0.5). Table 3 shows the predicted responses. No values are given for selection on BLUP-EBVs, since these will depend on details of the population (e.g., distant relatives) that are not considered here.

**Table 3:** **Predicted response for survival time in purebred and crossbred laying hens using individual selection, group selection, and selection based on relatives**.

**Selection method^a^**	***m*^b^**	**∆G_predicted_ Purebred**			**∆G_predicted_ Crossbred**		
		**Unrelated**	**HS**	**FS**	**Unrelated**	**HS**	**FS**
IS	1	9.7	12.6	15.5	−8.1	0.0	8.1
GS	1	9.6	16.1	22.1	10.5	17.5	24.1
SR	1		8.8	16.7		9.3	18.0
	10		19.1	30.4		21.5	35.0

a *IS is individual selection; GS is group selection; SR is selection based on relatives*.

b *m is number of groups per selection candidate. Response were predicted using ∆G = ιρσ_TBV_, where ι = 1. For each selection method, ρ was based on the Equations presented in Table 2. To predict ρ and σ_*TBV*_, genetic parameters for survival time were used (Ellen et al., 2008; Peeters et al., 2012) as shown in Table S1*.

In purebred laying hens, the covariance between DBV and SBV was positive. Therefore, for all selection methods, predicted response for survival time was positive, ranging from 8.8 through 30.4 days (Table 3). In crossbred laying hens, the covariance between DBV and SBV was moderately to strongly negative. Therefore, for individual selection response to selection was negative (−8.1 days), when selection candidates were kept with unrelated group mates and zero when selection candidates were kept with half sibs. This result implies that, for those group compositions, responses to BLUP-selection using Equation 10 will also be negative and around zero (compare Equations 11 and 6). For both purebreds and crossbreds, with a single group of related individuals, group selection resulted in the largest predicted response to selection. With ten groups of related individuals, selection based on relatives resulted in the largest predicted response to selection. For both purebred and crossbreds, and for all selection methods, using groups of full sibs resulted in the largest predicted response to selection. Note that, when accurate estimates of genetic parameters are available, selection on estimated total breeding values from Equation 12 is always equally good or better than any other selection method applied to the same population structure (Muir et al., 2013).

In conclusion, highest accuracies and responses to selection for socially-affected traits will be obtained using a population structure where individuals are kept in family groups.

## Empirical evidence of social genetic effects

### Estimated genetic parameters

Several studies have estimated genetic parameters for socially-affected traits. Table 4 gives an overview of the estimated heritabilities (*h*^2^) from a classical model, estimated total heritable variance relative to the phenotypic variance (*T*^2^) from a direct-indirect effects model, and the estimated genetic correlations between direct breeding values and social breeding values (*r_A_*).

**Table 4:** **Overview of genetic parameters using a classical model and a direct-indirect effects model**.

**Species**	**Trait**	**Classical model**	**Direct-indirect effects model**	
		***h*^2^**	***T*^2^**	***r_A_***
**CATTLE (*BOS TAURUS*)**				
	Feed lot growth rate^1^	0.06	2.01	0.69
	Social dominance^2^	0.12	0.01	−0.98
**COD (*GADUS MORHUA*)^3^**				
	Change in condition factor	0.13	0.22	−0.08 (n.s.)
	Dorsal fin erosion	0.01–0.83	0.48–1.29	0.30–0.78 (n.s.)
	Caudal fin erosion	0.06	0.43	0.21 (n.s.)
	Body weight	0.24–0.34	0.41–0.43	0.05–0.31 (n.s.)
**DEER MICE (*PEROMYSCUS MANICULATUS*)^4^**				
	Rearing rate	0.10	0.61	0.79
	Reciprocal latency to fight	0.05	0.56	0.86
**FOREST TREE (*EUCALYPTYS GLOBULUS*)^5^**				
	Diameter at breast height	0.34–0.42	0.05–0.08	~ −0.9
	Mycrospaerella leaf disease	0.41	0.67	0.8
**LAYING HENS (*GALLUS GALLUS*)**				
	Survival time, purebred^6^	0.07–0.10	0.15–0.19	−0.31 to 0.18 (n.s.)
	Plumage condition, purebred^7^	0.02–0.10	0.10–0.54	−0.38 to 0.16 (n.s.)
	Survival time, crossbred^8^	0.05–0.06	0.17–0.26	−0.83 to −0.37
	Early egg performance, crossbred^9^	NE	0.50–0.55	NE
**MINK (*NEOVISON VISON*)^10^**				
	Total bite mark score	0.23	0.61	0.90
**MUSSEL CULTURES (*MYTILUS GALLOPROVINCIALIS*)^11^**				
	Length	0.17	0.21	−0.09 (n.s.)
	Area	0.17	0.27	−0.30(n.s.)
**NILE TILAPIA (*OREOCHROMIS NILOTICUS*)^12^**				
	Harvest weight	0.31	0.32	−0.38
**PIGS (*SUS SCROFA*)**				
	Growth rate fattening^13^	0.20	0.59	0.24
	Growth rate fattening^14^	0.13	0.23	−0.02 (n.s.)
	Final body weight^15^	0.39	0.47	0.07 (n.s.)
	Back fat depth^15^	0.45	0.55	0.08 (n.s.)
	Muscle area^15^	0.29	0.31	−0.63 (n.s.)
	Growth suckling piglets^16^	0.07	0.15	−0.27 (n.s.)
	Androstenone^17^	0.61	0.75	0.24 (n.s.)
	(Net) Daily gain^18^	0.22–0.24	0.32–0.34	0.01
	Feed intake^18^	0.19	0.35	0.05
**RED DEER (*CERVUS ELAPHUS*)^19^**				
	Social dominance	0.10	0.03	−0.91
**SITKA SPRUCE (*PICEA SITCHENSIS*)^20^**				
	Diameter			−0.93
**QUAIL (*COTURNIX COTURNIX JAPONICA*)^21^**				
	Body weight	0.16	1.35	−0.24

1 Van Vleck et al., 2007, first 28 days of growth period;

2 Sartori and Mantovani, 2013;

3 Nielsen et al., 2014;

4 Wilson et al., 2009;

5 Costa e Silva et al., 2013;

6 Ellen et al., 2008;

7 Brinker et al., 2014;

8 Peeters et al., 2012;

9 Peeters et al., 2014;

10 Alemu et al., 2014b;

11 Brichette et al., 2001;

12 Khaw et al., 2014;

13 Chen et al., 2008;

14 Canario et al., 2010, d = 1;

15 Hsu et al., 2010;

16 Bouwman et al., 2010, model 4;

17 Duijvesteijn et al., 2012;

18 Bergsma et al., 2013;

19 Wilson et al., 2011;

20 Brotherstone et al., 2011;

21 *Muir et al., 2013; NE is not estimable; n.s. is not significant*.

In most populations, total heritable variance was greater than the ordinary additive genetic variance (*T*^2^ > *h*^2^). In two populations of trees, however, a strongly negative direct-social genetic correlation was found, causing total heritable variance to be smaller than additive genetic variance (Brotherstone et al., 2011; Costa e Silva et al., 2013). In those cases, there is strong heritable competition, and social interactions may decrease total heritable variation to zero (Costa e Silva et al., 2013). Moreover, for some traits competition is necessarily complete, so that there cannot be a response to selection. For example, in dyadic fighting contests, where the trait of interest is winning vs. loosing (1–0), a change in population mean is impossible since each contest has precisely one winner and one loser. Social effects models properly account for this by fitting a direct-indirect correlation of -1 and a total heritable variance of zero (Wilson et al., 2011; Sartori and Mantovani, 2013).

Table 4 shows that for most traits, social interactions had a substantial effect on the total heritable variation, explaining 6% through 98% of *T*^2^. For example, for survival time in laying hens showing cannibalism, social interactions explain 33% through 87% of the total heritable variation in survival time (Ellen et al., 2008; Peeters et al., 2012). The classical animal model suggests a genetic standard deviation of 27–44 days, whereas the direct-social effects model yields a standard deviation of the total breeding value of 50–65 days. In those cases, response to selection can be increased by taking into account social effects in the selection strategy.

There appears to be no systematic pattern in the direct-social genetic correlation (*r_A_*). For example, for bite mark score in mink *r_A_* was strongly positive, meaning that an individual that bites more (social effect) also attract more bites (direct effect) and *vice versa* (Alemu et al., 2014b). At first glance, biting in mink may seem similar to pecking in laying hens. Peeters et al. (2012) found a strongly negative *r_A_* for survival time in crossbred laying hens, indicating that individuals that live longer are more likely to be cannibalistic, i.e., lives longer at the expense of others. This is precisely opposite to the situation in mink. In quail, Muir (2005) also found a strong negative *r_A_* for growth indicating that birds that grew the fastest reduced the growth of other birds in the group due to strong negative social interactions.

### Selection experiments

Evidence of social genetic effects may also be obtained from selection experiments aiming to utilize such effects to generate response to selection. One of the first empirical studies used group selection for increased or decreased population size in randomly formed groups of flour beetles (Wade, 1976, 1977). In both directions, group selection was effective, even though groups were composed at random, whereas individual selection was not effective. Goodnight (1985) compared individual and group selection for leaf area in *Arabidopsis*. Leaf area responded to group selection, but not to individual selection. These results suggest the presence of social genetic effects (σ^2^*_A_S__* > 0), together with a negative direct-social genetic correlation.

Muir et al. (Craig and Muir, 1996; Muir, 1996) used group selection to improve survival and egg number of laying hens in multiple-bird cages. In their study, each sire family was housed as a group in nine-bird cages, and selected or rejected based on the performance of the group. The group-selected line kept in multiple-bird cages was compared with an unselected control line kept in single-bird cages. Mortality in the selected line decreased from 68% in generation 2–8.8% in generation 6. In generation 6, the mortality of the selected line was similar to that of the unselected control kept in single-bird cages (Muir, 1996). This rapid short-term response suggests a substantial social genetic variance in mortality. In the seventh generation, the selected line was compared with a control and a commercial line all kept in multiple bird cages. Hens of the selected line had a significantly better plumage condition than hens of the control and commercial line, whereas there was no significant difference in body weight (Craig and Muir, 1996).

In another experiment, Muir (2005) selected for TBV among individuals kept in groups of 16 members to improve 43-day body weight in Japanese quail. Individuals of the first two generations were used to estimate genetic parameters. In subsequent generations, parents were selected either on TBV (C-BLUP) or on direct EBVs only (D-BLUP; Muir, 2005). After 6 generations, C-BLUP resulted in a significant improvement of body weight, whereas D-BLUP resulted in a non-significant decrease in body weight. Furthermore, selection using C-BLUP resulted in a slight decrease in mortality, whereas D-BLUP resulted in an increase in mortality. These results suggest presence of social genetic effects and a negative direct-social genetic correlation.

Later on, Muir et al. (2013) used multi-level selection on classical BLUP-EBVs to improve 43-day body weight in Japanese quail. They compared two experimental set ups; individuals were either kept in family groups or in groups with unrelated individuals. After 18 mini generations (MG; five MG is one generation), responses were positive with family groups, resulting in a regression coefficient of 1.30 g/MG, whereas responses were much smaller with unrelated groups (regression coefficient of 0.13 g/MG). Furthermore, a significant difference in mortality was found, yielding the lowest mortality in family groups (6.6 vs. 8.5% in unrelated groups). Again, results indicate presence of social genetic effects, and agree with the theoretically expected effect of relatedness on response to selection (see above).

**Figure 1:**
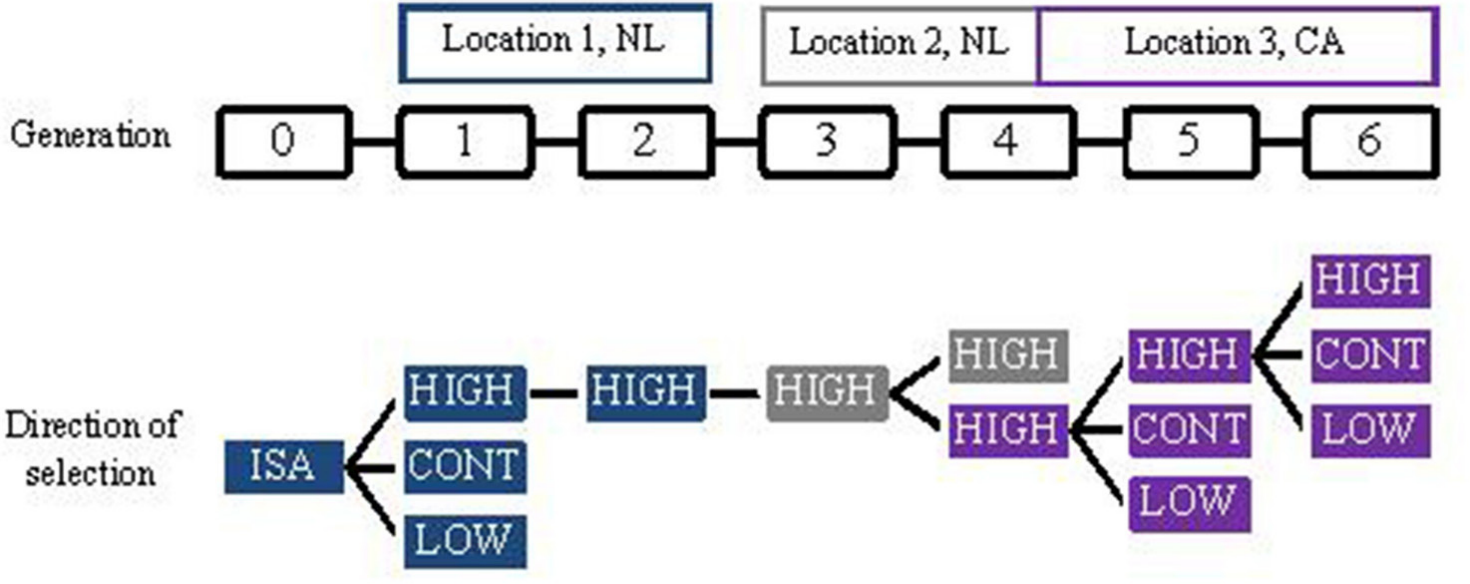
**Design of the selection experiment**.

Ellen et al. (2013, in prep) investigated the potential to select against mortality due to cannibalism in laying hens, within the ordinary commercial operations of a laying breeding company, where selection candidates are kept individually. In total, six generations were selected. In each generation, individually housed selection candidates were selected based on survival time of relatives kept in family groups. Relatives had intact beaks and were kept with 4 or 5 sibs in traditional battery cages under commercial circumstances. Figure 1 gives an overview of the selection design. For generations 1, 5, and 6, selection candidates were selected in two directions, high (HIGH) and low (LOW) survival. Remaining selection candidates were used to breed a control group (CONT). For generation 2 through 4, selection candidates were selected only to breed HIGH, and there was no CONT present. Because hens of the six generations were kept at different locations (Figure 1), it was not possible to compare hens of HIGH across generations. Table 5 shows the expected and realized responses. Because information on survival becomes available late in life, in ordinary commercial operation individuals had to be mated when information on survival was very limited, resulting in a low selection intensity and expected responses (Ellen et al., 2014). In generation 1, 5, and 6, the realized difference in survival days between HIGH and LOW ranged from 26 to 29 days. Difference in survival days between HIGH and CONT was 13 and 19 days in generation 1 and 6, respectively, whereas the difference was −12 days in generation 5. On average, these realized differences agree with the theoretical expectation. These results show that selection against mortality due to cannibalism is feasible under ordinary commercial circumstances, but also that it is difficult to achieve high intensities of selection. Moreover, they illustrate that mortality due to cannibalism is very sensitive to changes in the environment (e.g., stocking density, light intensity, climate).

**Table 5:** **Expected and realized responses for survival time in laying hens using selection based on relatives**.

**Location**	**Generation**	**ι^1^**	**ρ*TBV*,*SR*^2^**	**∆*G_expected_*^3^ (days)**	**∆*G_expected_* (days)**	
					**HIGH vs. CONT**	**CONT vs. LOW**
1	1	0.14	0.3	1.7	13	16
	2	0.09	0.3	1.3		
2	3	0.52	0.2	3.5		
	4	0.21	0.3	1.9		
3	4	0.25	0.3	4.0		
	5	0.32	0.3	5.0	−12	40
	6	0.32	0.3	2.8	19	7

*^a^ι = S_P_/σ_P_; ^b^$\rho_{TBV,SR}=r_{rel}\eta /\sqrt{\tau +\left(1-\tau \right)/mn}$, where η = σ_TBV_/σ_TPV_, τ = rσ^2^_TBV_/σ^2^_TPV_, and m = 1; ^c^∆G_expected_ = ιρ_TBV,SR_σ _TBV_, where ι and ρ refer to parents*.

Selection also changed the physiology and behavior of birds. In generation 2, hens of HIGH showed less fear-related behavior than hens from the founder line (Bolhuis et al., 2009). This was confirmed both in young (before cannibalism develops) and in adult birds using sibs of generation 4 (Rodenburg et al., 2009a,b; Nordquist et al., 2011; de Haas et al., 2012). In generation 2, hens of HIGH had higher whole-blood serotonin concentrations and a lower platelet serotonin uptake velocity than hens of the founder line, indicating differences in functional activity of the serotonergic system (Bolhuis et al., 2009). Again, results were confirmed in sibs of generation 4. Moreover, HIGH hens of generation 4 showed dopaminergic and noradrenergic changes in two brain areas, the arcopallium (Kops et al., 2013) and the nidopallium caudolaterale (Nordquist et al., 2013). These results are in line with the proposed role of serotonergic and dopaminergic activity in feather pecking behavior (Van Hierden et al., 2004). Furthermore, sibs of generation 4 showed a reduced stress response to manual restraint and less comb and toe lesions, indicating lower levels of aggression and cannibalism (Rodenburg et al., 2009a).

Camerlink et al. (2013) investigated the effect of one generation of selection on diverging social breeding values for growth rate in pigs. In commercial pigs, behaviors such as aggressive attacks, tail biting and other injurious oral manipulation of group mates, may profoundly affect welfare and productivity. In their selection experiment, dams and sires with the most extreme (HIGH and LOW) EBVs for social genetic effects for growth during the finishing phase were selected to create the next generation, while DBVs were kept the same for both populations. In the offspring, the estimated contrast for social genetic effects was 14 g ADG (Camerlink et al., 2013, 2014b). After weaning, offspring were housed in pens of six unrelated individuals. Surprisingly, both populations did not differ in growth during the finishing phase, which could be due to the relatively small contrast in EBVs. Camerlink et al. (2014a) suggested, however, that this unexpected result might also be due to the fact that measures were taken to limit harmful behavior to an acceptable level to safeguard the welfare of the experimental animals, which may have reduced the effects of this harmful behavior on growth rate (Camerlink et al., 2012). Even though there was no effect on growth rate, systematic differences in behavior were found between both groups. HIGH pigs showed less unilateral biting and less ear biting (Camerlink et al., 2014b). They also had a lower usage of jute sacks, and inflicted less tail damage, whereas no effects on general activity were found (Camerlink et al., 2014b). Moreover, HIGH pigs showed less aggression at reunion with familiar group mates after a 24-h regrouping test in which they were confronted with unfamiliar conspecifics (Camerlink et al., 2013), which is likely related to differences in stress-sensitivity rather than aggressiveness *per se*, as no differences in aggression during mixing or in body lesion scores were found. In line with this, these HIGH pigs tended to respond less fearfully and stressed to novel and challenging situations (Reimert et al., 2014a), already during the piglet stage (Reimert et al., 2013) and they had lower leukocyte, lymphocyte and haptoglobin concentrations than LOW pigs (Reimert et al., 2014b). These behavioral and physiological data indicate that selection on high SBV for growth can result in pigs that show less harmful biting behavior, such as tail biting, and are possibly less fearful and better capable of handling stressful situations (Camerlink et al., 2013; Reimert et al., 2014a,b).

## Application

Livestock are nowadays more frequently kept in (larger) groups, resulting in an increase in social interactions between individuals. Moreover, treatments to limit the consequences of adverse social interactions, such as beak trimming in poultry and tail docking in pigs, will probably be banned in the future (at least in EU countries), so that the negative effects of social interactions will likely increase unless action is taken to avoid that. Actions are needed to prevent or diminish the negative effects of social interactions. In this review, we have shown that many traits show genetic variation in social effects. Moreover, we have reviewed selection methods for socially-affected traits, showing that methods exist that utilize the social genetic variation for genetic improvement. Thus, the genetic variation and selection tools required for genetic improvement of socially-affected traits are available, indicating that genetic solutions are feasible in principle. Nevertheless, successful application in commercial breeding programs faces a number of challenges, some of which we review below.

### Accuracy of EBVs

For commercial livestock breeding, it is most important to estimate accurate breeding values. When the objective is to separately estimate direct and social breeding values, rather than only the total breeding value, genetic parameters for direct and social effects are required. Genetic parameters for direct and social effects cannot be estimated when group members are equally related (i.e., all full sibs or half sibs). The optimal design for estimating direct and social genetic parameters has groups composed of two families. Moreover, the number of groups, rather than the number of individuals, is the key parameter determining accuracy of the estimated variance components. Bijma (2010a) showed that ~250–500 groups are needed.

The optimal group-composition for estimating direct and social genetic parameters differs from the optimal group-composition for estimating the TBV and maximizing response to selection. Accuracies of estimated TBVs are maximized when using groups composed of families (Griffing, 1976; Ellen et al., 2007; Muir et al., 2013). However, direct and social genetic parameters cannot be estimated from such designs (Bijma, 2010a). Groups composed of two families probably also yield good accuracy of estimated TBVs, certainly better than groups composed at random, but this has not been investigated thoroughly.

When interest is merely in the TBV, rather than in separate breeding values for direct and social effects, there appears to be less conflict between estimation of genetic parameters and breeding values. In this case, groups consisting of complete families can be used to estimate both the total additive genetic variance and TBVs of selection candidates. This can be achieved by fitting a classical animal model ignoring social genetic effects, as the additive genetic variance and EBVs from this model will refer to the TBV (Peeters et al., 2013, 2014). However, this will only work well if groups indeed consist of complete families.

In commercial pig production, the number of groups is often limited, and separate rooms within a barn often consist of a limited number of groups. This design makes it challenging to estimate accurate genetic parameters and breeding values for direct and social effects, and validation is difficult (Duijvesteijn, 2014). Group composition is not always recorded accurately in pig production. This will affect both the EBV of the individual of interest, and the EBVs of its group mates, and may lead to exclusion of entire groups. Moreover, group composition often changes over time because individuals are regrouped to create homogeneous groups, so as to avoid penalties when delivering pigs to the slaughter house. This creates serious problems for the breeding value estimation. For example, it is unclear which individuals to include as social partners in the model, and how to weigh those individuals. In principle, one could weigh social effects of group mates on the focal individual by the time both individuals spent together in the group. However, regrouping of individuals is often not at random, but based on individual traits that are partly genetic. Hence, simply weighing the social incidence matrix by the time both individuals spent together may therefore bias the breeding value estimation (personal observations in simulated data). Hence, when pig breeders aim to improve social genetic effects, regrouping should be avoided in breeding herds.

Other livestock species, such as dairy cattle and broilers, are regularly kept in one large group per farm. In this design, it is not (yet) possible to estimate genetic parameters for direct and social effects (see also paragraph about social genetic effects in large groups). This occurs because direct and social genetic parameters are not statistically identifiable when fitting a fixed effect for the farm (Cantet and Cappa, 2008). Consequently, it is unknown whether social interactions are important in dairy cattle and broilers.

In laying hens, small groups of sibs are used to evaluate roosters (so-called recurrent tests). Though direct and social genetic parameters cannot be estimated from this design, the design is ideal for the estimation of TBVs and probably also for the estimation of total genetic variance (Peeters et al., 2013, 2014). Hence, ordinary recurrent tests in laying hen breeding programs implicitly includes the social effects in the EBVs in an optimum manner, even though they are not explicitly modeled.

When the data contain repeated observations, presence of permanent environmental effects may complicate the estimation of genetic parameters and breeding values for social effect. In beef cattle, for example, permanent environmental effects may cause overestimation of breeding values for maternal effect when information on paternal additive genetic relationships is limited. To our knowledge, the impact of permanent environmental effects on the genetic analysis of socially-affected traits has not been investigated.

### Purebred vs. crossbred populations

In commercial pig and poultry farming, crossbred populations are used. So far, selection experiments to improve socially-affected traits in laying hens and pigs have focused on purebred populations. Efficient improvement of socially-affected traits in crossbred populations based on data from purebred populations requires a purebred-crossbred genetic correlation (*r_pc_*) close to one. When *r_pc_* is small to moderate, crossbred information is needed. So far, however, *r_pc_* for socially-affected traits is unknown. Results in laying hens suggest that socially-affected traits in crossbreds may differ considerably from those in purebreds. Peeters et al. (2012) found that average survival time in crossbreds was much lower than in purebreds, while social genetic effects were much larger in crossbreds. Furthermore, they found a direct genetic correlation between both crosses of almost 1, but a social genetic correlation of only 0.41. When the social genetic correlation between both lines is only 0.41, it is mathematically impossible that both purebred-crossbred social genetic correlations are near one. Thus, results of Peeters et al. (2012) suggest that *r_pc_* is lower for social effects than for direct effects, indicating a greater need for crossbred data when selecting for socially-affected traits. In principle, one could estimate *r_pc_* to decide on the need for crossbred information. However, unless data are available already, the amount of data required to accurately estimate *r_pc_* is not very different from the amount required to select based on crossbred information, particularly when using genomic selection (Bijma and Bastiaansen, in press). Hence, it is probably better to start breeding for crossbred performance immediately, and estimate *r_pc_* once sufficient data has been collected.

### Environment

As with any trait, expression of socially-affected traits will depend on the environment, and genotype-by-environment (GxE) interaction may occur. Whether GxE-interactions are greater for socially-affected traits than for other traits is unknown at present. Cannibalism in laying hens is very sensitive to environmental conditions. Ellen et al. (in preparation), for example, found a 20% difference in survival when birds of the same generation were kept at two different locations. Whether such large differences imply substantial GxE-interaction is unclear. GxE-interaction due to differences between purebred and crossbred environments would reduce *r_pc_*, but this can be resolved by selection based on crossbred information. GxE-interaction between different commercial environments, however, would reduce additive genetic variance expressed in the overall environment, restricting response to selection irrespective of the data used for selection.

The expression of social interactions might also depend on early life experiences. In laying hens, incubation and rearing conditions substantially affect feather pecking and cannibalism (reviewed in van de Weerd and Elson, 2006; Rodenburg et al., 2008). Ellen et al. (in preparation) found an 18% difference in survival between different batches of hens kept in the same environment. These batches were hatched at different weeks. In pigs, early isolation changed behavioral, neuroendocrine, and immune regulation, which can have negative consequences for health and welfare later in life (Kanitz et al., 2004). Therefore, to improve socially-affected traits, it is important to also consider early life experience.

Within a group, however, there can be different social interactions. Individuals tend to behave different toward strangers than to familiar (sibs or reared in the same group) individuals, also known as kin recognition (Hamilton, 1964). When groups consist of both sibs and random individuals, genetic parameter estimation using the direct-indirect effects model as shown in Bijma et al. (2007a) can result in biased estimates of social genetic effects and can yield suboptimal response to selection (Alemu et al., 2014a). Both in pigs and fish it was found that kin recognition explained a substantial part of the phenotypic variation, after correcting for group and family effects (Duijvesteijn, 2014; Khaw et al., 2014). However, when social genetic effects differ between kin and non-kin, it is not (yet) possible to estimate those genetic parameters (Alemu et al., 2014a). Further studies are needed to disentangle the social genetic effect for kin and non-kin.

### Social genetic effects in large groups

So far, estimation of genetic parameters and selection experiments focused on relatively small group sizes. Small group sizes have been used for several reasons. For estimation of genetic parameters, small groups are preferred (1) because in small groups it is a reasonable assumption that all group members interact with each other; and (2) because accurate estimation of social genetic parameters requires data on many groups (Bijma, 2010a). For estimation of breeding values and selection, small groups have been used (1) to have related individuals in a group (either full sibs or half sibs); (2) to have at least one group of relatives per family. Both lead to increased accuracy of the selection method. When groups are large, it is unclear which individuals interact with each other, and the number of groups will be small resulting in inaccurate breeding values. So far, no experiments or analysis have been done to improve socially-affected traits in large groups.

We see two opportunities to genetically improve socially-affected traits in large groups. First, selection decisions can be based on breeding values estimated from data on small groups. This approach will be successful only when the correlation between total breeding values in small *vs*. large groups is reasonably close to one. Whether that is the case is an empirical question. In laying hens, for example, feather pecking and mortality due to cannibalism are more problematic in larger groups (e.g., Nicol et al., 1999; Bilčik and Keeling, 2000; Lay et al., 2011). Furthermore, spreading of social interactions due to social learning might be more pronounced in larger groups, but larger groups may also show greater social tolerance (Turner et al., 2001; Zimmerman et al., 2006). Second, one can attempt to estimate genetic parameters and breeding values from data on large groups, or even a single group. This requires that the individuals that interact with each other are identified. In a forest, for example, social genetic effects can be estimated by using the inverse of the distance between two trees in the incidence matrix for social effects (Muir, 2005). When the location of individuals in large groups can be traced sufficiently precise, for example with sensor technology, similar approaches may be feasible in livestock. Such systems are not available at present, but the basic technology exists.

## Future directions

In this part, we will describe some future developments, which also hold promise for social genetic effects models.

### Genomic selection

Genomic selection is currently being implemented in livestock breeding. Genomic selection has the greatest impact for traits that are: difficult to measure, cannot be measured on the selection candidates, are measured late in life, or have low heritability (Meuwissen et al., 2001; Muir, 2007). Improvement of socially-affected traits using genomic selection would be promising for a number of these reasons (Muir et al., 2014). For mortality due to cannibalism in laying hens, genomic selection would solve the problem of low intensities of selection that occurs in traditional schemes because information becomes available late in life (see Table 5). On the one hand, genomic selection methods could be extended to explicitly include social genetic effects; i.e., to estimate both direct and social genomic EBVs. For this purpose, the additive genetic relationship matrix (see below Equation 12) could be replaced with a genomic relationship matrix, an approach known as “Genomic BLUP” (Strandén and Garrick, 2009), or with a relationship matrix combining pedigree and genomic data, known as the H-matrix (Legarra et al., 2009). The use of genomic information may help to solve identifiability issues, since pairs of full sibs no longer all have the same relationship. A challenge will be to design a reference population that can be used for genomic selection of socially-affected traits. However, so far it is unknown what the optimal design of the reference population is (i.e., group structure, number of groups, relatedness within a group). An alternative is to use family groups and estimate total genomic breeding values. For example, in recurrent tests in laying hens where crossbred offspring are kept in sire-family groups, genotyping the fathers and fitting an ordinary genomic selection model would yield genomic estimates of total breeding values, rather than direct breeding values. A similar approach could be used for tail-biting in pigs, where crossbred offspring could be kept in full-sib groups.

### Social genetic effects and diseases

In this review, social interactions have implicitly been interpreted as behavioral interactions. However, also infectious disease traits, represent socially-affected traits. The disease status of an individual is affected both by the individual's susceptibility to the disease (direct effect) and by the infectivity of its social partners (social effect; Lipschutz-Powell et al., 2012). Classical genetic analyses of disease data focused on individual susceptibility (Lipschutz-Powell et al., 2012). Recently, researchers started to model infectious diseases using social genetic effects models. Anche et al. (2014) showed that the individual's breeding value for *R*_0_ (*R*_0_ determines risk and severity of infectious diseases) is a function of its own allele frequency for susceptibility and infectivity and of the population average susceptibility and infectivity. Again, relatedness between interacting individuals is an important component, resulting in increased response in *R*_0_. This work, therefore, suggests that breeders can considerably increase response to selection in infectious disease traits by collecting disease data from family groups. This is the case even when there is no genetic variation in infectivity, since also genetic variation in susceptibility generates social genetic effects (see Anche et al., 2014, for details). Empirical studies are needed to confirm theoretical expectations. These approaches could also lead to novel insights applicable in the field of breeding animals for group housing.

## Conclusion

Social interactions are important for livestock genetic improvement. Applying a selection method that targets both direct and social effects will be a key factor to improve welfare and productivity of livestock simultaneously. There is growing evidence that methods are effective for animals kept in small groups. Challenges are in the application in commercial livestock breeding programs, for example in populations consisting of large groups.

## Author contributions

Esther D. Ellen wrote and prepared the manuscript for submission. T. Bas Rodenburg, J. Elizabeth Bolhuis, Egbert F. Knol, and Gerard A. A. Albers were involved in the discussion of the manuscript and reviewed the manuscript. T. Bas Rodenburg, J. Elizabeth Bolhuis, Irene Camerlink, Naomi Duijvesteijn, William M. Muir, and Piter Bijma wrote and reviewed the manuscript. Katrijn Peeters, Inonge Reimert, Ewa Sell-Kubiak, Johan A. M. van Arendonk, and Jeroen Visscher reviewed the manuscript.

### Conflict of interest statement

The authors declare that the research was conducted in the absence of any commercial or financial relationships that could be construed as a potential conflict of interest.
