# Supplementary material for: The prospects of selection for social genetic effects to improve welfare and productivity in livestock
Source: Front Genet. 2014 Nov 11;5:377. doi: 10.3389/fgene.2014.00377 (PMC4227523; doi:10.3389/fgene.2014.00377)
Supplement: Supplementary file 1 [file Table1.DOCX]

**Appendix I. Estimated genetic parameters for survival time in laying hens**

Here, we summarize the estimated genetic parameters for survival time in purebred ([line W1; Ellen et al., 2008](#_ENREF_38)) and crossbred ([cross WB*W1; Peeters et al., 2012](#_ENREF_75)) laying hens (Table S1).

**Table S1.** Genetic parameters for survival time in purebred and crossbred laying hens

| Parameter^1^ | Purebred | Crossbred |
| --- | --- | --- |
| $\sigma_{e}^{2}$ | 10,608 | 15,655 |
| $\sigma_{A_{D}}^{2}$ | 915 | 997 |
| $\sigma_{A_{S}}^{2}$ | 134 | 767 |
| $\sigma_{A_{DS}}$ | 62 | -726 |
| $\sigma_{TBV}^{2}$ | 2,493 | 3,544 |
| $\sigma_{g}^{2}$ | 922 | 2,379 |
| $\sigma_{P}^{2}$ | 13,435 | 21,455 |
| $\sigma_{TPV}^{2}$ | 18,423 | 33,323 |
| $\sigma_{P_{grp}}^{2}$ | 4,985 | 8,478 |
| $\eta^{2}$ | 0.14 | 0.11 |
| *n* | 4 | 4 |

^1^ $\sigma_{e}^{2}$ is residual variance; $\sigma_{A_{D}}^{2}$ is direct genetic variance; $\sigma_{A_{S}}^{2}$ is social genetic variance; $\sigma_{A_{DS}}$ is direct-social genetic covariance; $\sigma_{TBV}^{2}$ is total heritable variation: $\sigma_{TBV}^{2}=\sigma_{A_{D}}^{2}+2\left( n-1 \right)\sigma_{A_{DS}}+\left( n-1 \right)^{2}\sigma_{A_{S}}^{2}$; $\sigma_{g}^{2}$ is non-genetic group variance; $\sigma_{P}^{2}$ is phenotypic variance: $\sigma_{P}^{2}=\sigma_{A_{D}}^{2}+\sigma_{E_{D}}^{2}+\left( n-1 \right)\left( \sigma_{A_{S}}^{2}+\sigma_{E_{S}}^{2} \right)+r\left[ 2\left( n-1 \right)\sigma_{A_{DS}}+\left( n-1 \right)\left( n-2 \right)\sigma_{A_{S}}^{2} \right]$, $\sigma_{E_{D}}^{2}+\left( n-1 \right)\sigma_{E_{S}}^{2}=\sigma_{e}^{2}$, *r* = 0.5; $\sigma_{TPV}^{2}$ is total phenotypic variance: $\sigma_{TPV}^{2}=\sigma_{P_{D}}^{2}+2\left( n-1 \right)\sigma_{P_{DS}}+\left( n-1 \right)^{2}\sigma_{P_{S}}^{2}$, see Ellen et al. ([2007](#_ENREF_35)) for the derivation of $\sigma_{TPV}^{2}$; $\sigma_{\bar{P}_{grp}}^{2}$ is phenotypic variance among groups: $\sigma_{\bar{P}_{grp}}^{2}=\left\{ \sigma_{P}^{2}+2\left( n-1 \right)Cov\left( P_{i},P_{j} \right)+\left( n-1 \right)\left[ \sigma_{P}^{2}+\left( n-2 \right)Cov\left( P_{i},P_{j} \right) \right] \right\}/{n^{2}}$ ([Ellen et al., 2007](#_ENREF_35)), *r* = 0.5; $\eta^{2}$ is an analogy of the heritability: $\eta^{2}={\sigma_{TBV}^{2}}/{\sigma_{TPV}^{2}}$ ; *n* is number of group members.
